# Meta-analysis of randomized phase II trials to inform subsequent phase III decisions

**DOI:** 10.1186/1745-6215-15-346

**Published:** 2014-09-03

**Authors:** Danielle L Burke, Lucinda J Billingham, Alan J Girling, Richard D Riley

**Affiliations:** Medical Research Council Midland Hub for Trials Methodology Research, School of Health and Population Sciences, University of Birmingham, Edgbaston, Birmingham B15 2TT UK; Cancer Research UK Clinical Trials Unit, University of Birmingham, Edgbaston, Birmingham B15 2TT UK; School of Health and Population Sciences, University of Birmingham, Edgbaston, Birmingham B15 2TT UK

**Keywords:** Meta-analysis, Phase II and III, Prediction, Heterogeneity, Bayesian

## Abstract

**Background:**

If multiple Phase II randomized trials exist then meta-analysis is favorable to increase statistical power and summarize the existing evidence about an intervention's effect in order to help inform Phase III decisions. We consider some statistical issues for meta-analysis of Phase II trials for this purpose, as motivated by a real example involving nine Phase II trials of bolus thrombolytic therapy in acute myocardial infarction with binary outcomes.

**Methods:**

We propose that a Bayesian random effects logistic regression model is most suitable as it models the binomial distribution of the data, helps avoid continuity corrections, accounts for between-trial heterogeneity, and incorporates parameter uncertainty when making inferences. The model also allows predictions that inform Phase III decisions, and we show how to derive: (i) the probability that the intervention will be truly beneficial in a new trial, and (ii) the probability that, in a new trial with a given sample size, the 95% credible interval for the odds ratio will be entirely in favor of the intervention. As Phase II trials are potentially optimistic due to bias in design and reporting, we also discuss how skeptical prior distributions can reduce this optimism to make more realistic predictions.

**Results:**

In the example, the model identifies heterogeneity in intervention effect missed by an I-squared of 0%. Prediction intervals accounting for this heterogeneity are shown to support subsequent Phase III trials. The probability of success in Phase III trials increases as the sample size increases, up to 0.82 for intracranial hemorrhage and 0.79 for reinfarction outcomes.

**Conclusions:**

The choice of meta-analysis methods can influence the decision about whether a trial should proceed to Phase III and thus need to be clearly documented and investigated whenever a Phase II meta-analysis is performed.

**Electronic supplementary material:**

The online version of this article (doi:10.1186/1745-6215-15-346) contains supplementary material, which is available to authorized users.

## Background

Phase III trials are rigorous evaluations of an intervention (such as a new drug or surgical technique), and are typically protocol-driven with large patient numbers, appropriate statistical power, and a suitable trial design and analysis plan. However, the decision to initiate a Phase III trial for a particular intervention is not straightforward and depends on many factors, such as costs, risks (to the trial funders and patients), and practicalities such as patient recruitment [1]. Perhaps the most pivotal factor is the intervention's likely effectiveness. Clearly, the more likely an intervention is to succeed, the more likely funders will risk investment in a Phase III trial. To this end, before initiation of a Phase III trial funders will consider the existing evidence about an intervention's potential benefit, for example from earlier Phase trials.

The initial estimate of the intervention effect often arises from a Phase II randomized trial. These typically contain small patient numbers or events, and give an imprecise intervention effect estimate with a wide 95% confidence interval. However, sometimes multiple Phase II trials are conducted, for example in slightly different patient groups or by different (or competing) researchers (or companies) working on the same or similar interventions. In this situation, a meta-analysis is useful to increase statistical power [2] by combining the statistical estimates (such as odds ratios (ORs)) from the multiple trials and thereby summarizing the intervention effect based on the current evidence [3]. It is well established that a meta-analysis of Phase III randomized trials is influential towards deciding whether a particular intervention is used in clinical practice. However, there has been little consideration of methods for meta-analysis of Phase II trials, and how this approach might inform whether a Phase III trial should be initiated.

In this article we describe the key statistical issues when performing a meta-analysis of Phase II randomized trials, as motivated by a real example in acute myocardial infarction [4]. We show how Phase II meta-analysis results can be used to predict the potential intervention effect in a subsequent Phase III trial [5], and we explain why such predictions might be misleading unless between-trial heterogeneity and its estimation uncertainty are acknowledged. As Phase II trial results are particularly prone to optimism in the intervention effect, we also consider how to incorporate realistic or skeptical clinical beliefs about the size of the intervention effect [6]. The sensitivity of the meta-analysis estimates and inferences to the choice of prior distribution for the between-trial variance parameter is also explored [7]. We draw on previous discussions about the interpretation of meta-analysis [5, 8], more appropriate modelling of binomial data in meta-analysis [9], the derivation of prediction intervals for intervention effects in new trials [5], and the need to consider new trials in the context of previous meta-analyses [10]. We begin by outlining a motivating example of Phase II trials of thrombolytic therapy, and then introduce key statistical methods and issues with application to the example. We then consider an extension to deal with potential optimism and bias, and conclude with some discussion.

## Methods

In this section we introduce a motivating example, and then describe statistical methods for meta-analysis of Phase II trials.

### Motivating example: Phase II trials of bolus thrombolytic therapy for acute myocardial infarction

In patients with acute myocardial infarction, thrombolytic therapy aims to reduce mortality and restore normal blood flow by dissolving clots in blood vessels [11]. Eikelboom *et al*. [4] conducted a fixed-effect meta-analysis of nine Phase II trials (Table 1) that evaluated the efficacy of bolus thrombolytic therapy versus standard infusion therapy for the in-hospital treatment of acute myocardial infarction [11–19]. Two binary adverse event outcomes of interest were reinfarction and intracranial hemorrhage (ICH). Reinfarction is the clinical term given to a recurrence of a myocardial infarction (MI) that occurs within 28 days of an incident of a MI [20]. ICH is the accumulation of blood within the cranial vault and can lead to neurological dysfunction, elevation of intracranial pressure, and death [21]. For each outcome, Eikelboom *et al*. [4] compare their meta-analysis of these Phase II trials with a separate meta-analysis of six subsequent Phase III trials [22–27] (Table 1) to study if, in retrospect, they were in agreement.

**Table 1:** **Phase II and Phase III randomized trials of bolus versus infusion thrombolytic therapy for acute myocardial infarction**

Trial name, year published	Length of follow-up	Outcome	Sample size, N		Number of events (%)	
			Bolus	Infusion	Bolus	Infusion
**Phase II trials**						
RAPID [11] 1995	30 days	ICH	452	154	1 (0.2)	4 (2.6)
		Reinfarction			20 (4.4)	7 (4.5)
RAPID-II [12] 1996	35 days	ICH	169	155	2 (1.2)	3 (1.9)
		Reinfarction			8 (4.7)	7 (4.5)
Kawai *et al*. [13] 1997	7 days	ICH	97	102	0 (0)	1 (1.0)
		Reinfarction			4 (4.1)	7 (6.9)
Vanderschueren *et al*. [14] 1997	Hospital stay	ICH	50	52	0 (0)	0 (0)
		Reinfarction			5 (10.0)	7 (13.4)
BASE [15] 1998	Hospital stay	ICH	139	53	2 (1.4)	0 (0)
		Reinfarction			9 (6.5)	1 (1.9)
DOUBLE [16] 1998	30 days	ICH	224	237	2 (0.9)	1 (0.4)
		Reinfarction			5 (2.2)	12 (5.1)
lnTIME [17] 1998	30 days	ICH	478	124	0 (0)	1 (0.8)
		Reinfarction			9 (1.9)	8 (6.5)
TIMI 10B [18] 1998	30 days	ICH	540	316	9 (1.7)	6 (1.9)
		Reinfarction			28 (5.2)	18 (5.7)
TIMIKO [19] 1998	30 days	ICH	350	268	1 (0.3)	3 (1.1)
		Reinfarction			11 (3.1)	9 (3.4)
**Phase III trials**						
INJECT [22] 1995	35 days	ICH	2992	2994	23 (0.8)	11 (0.4)
		Reinfarction			150 (5.0)	162 (5.4)
COBALT [23] 1997	30 days	ICH	3585	3584	40 (1.1)	29 (0.8)
		Reinfarction			140 (3.9)	147 (4.1)
GUSTO III [24] 1997	30 days	ICH	10138	4921	92 (0.9)	43 (0.9)
		Reinfarction			426 (8.7)	207 (2.0)
BIRD [26] 1998	30 days	ICH	1196	1212	9 (0.8)	9 (0.7)
		Reinfarction			-	-
ASSENT-2 [25] 1999	30 days	ICH	8461	8488	79 (0.9)	80 (0.9)
		Reinfarction			347 (4.1)	325 (3.8)
lnTIME-II [27] 1999	30 days	ICH	10051	5027	114 (1.1)	31 (0.6)
		Reinfarction			-	-

ICH, intracranial hemorrhage.

**Figure 1:**
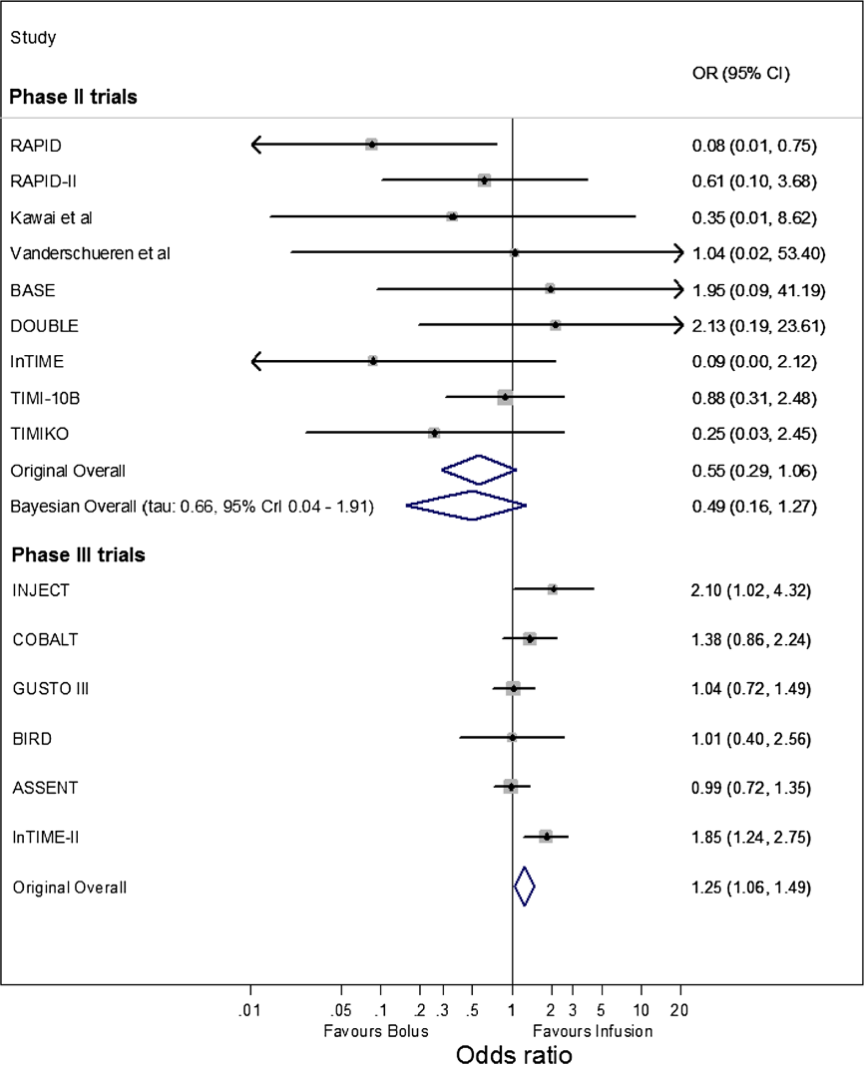
**Meta-analysis of Phase II and Phase III trials for ICH.** 'Original Overall' is the original summary results from the Phase II and Phase III fixed effect meta-analyses reported by Eikelboom *et al.* [4]; 'Bayesian Overall' is the summary result from a Bayesian random-effects logistic regression meta-analysis for the Phase II trials (see model 2), with 'tau' the estimated between-trial standard deviation. ICH, intracranial hemorrhage [11–19, 22–27].

**Figure 2:**
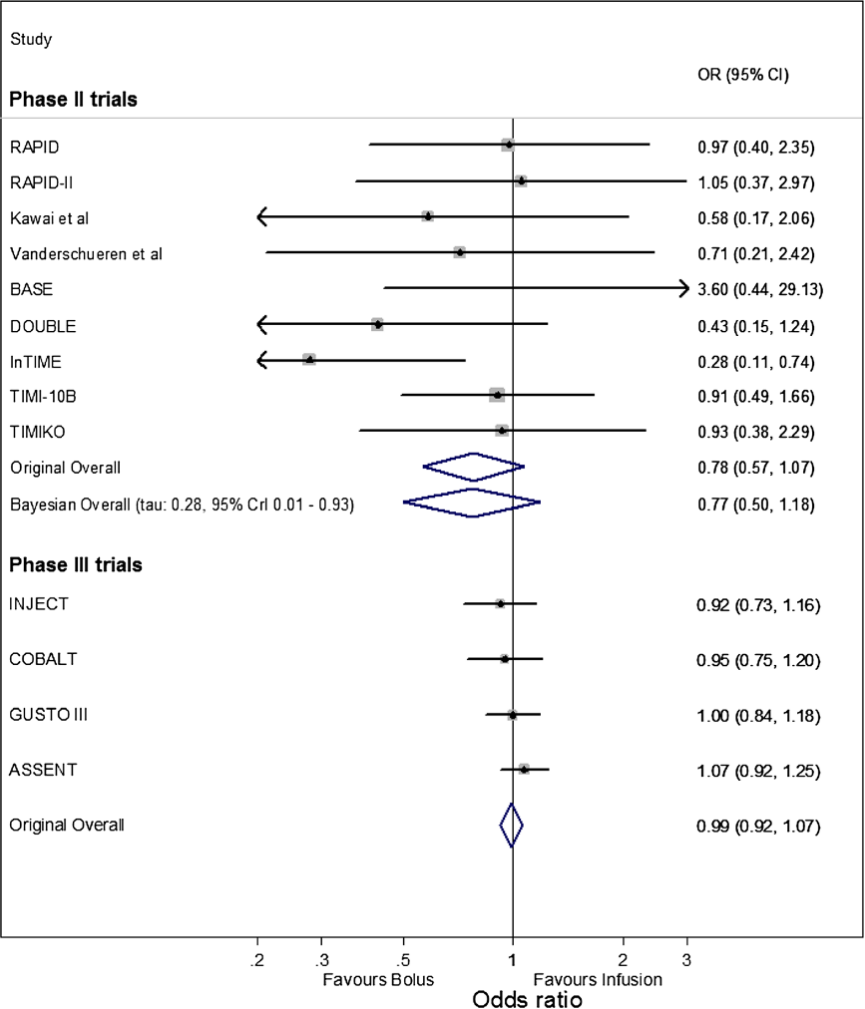
**Meta-analysis of Phase II and Phase III trials for reinfarction.** 'Original Overall' is the original summary results from the Phase II and Phase III fixed-effect meta-analyses by Eikelboom *et al.* [4]; 'Bayesian Overall' is the summary result from the Bayesian random-effects logistic regression meta-analysis for the Phase II trials (see model 2), with 'tau' the between-trial standard deviation. The data for reinfarction was not available for two of the six Phase III trials [11–19, 22–25].

The forest plots summarizing the OR estimate and 95% confidence interval for each included trial and the overall meta-analysis results are shown in Figure 1 for ICH and Figure 2 for reinfarction. The summary ORs obtained by Eikelboom *et al*. [4] appear similar for the Phase II and Phase III meta-analyses for reinfarction. However, for ICH the summary ORs are in opposite directions for the Phase II trials (OR: 0.55 with 95% CI 0.29 to 1.06) and Phase III trials (OR: 1.25 with 95% CI 1.06 to 1.49). Therefore, it might appear that the Phase II trials were a poor indication of how the intervention would perform in subsequent Phase III trials. Eikelboom *et al*. [4] suggest the discrepancy may be due to differences in patient populations and therapy intensity, alongside potential design and reporting biases in the Phase II trials.

In this article we evaluate this apparent conflict further by considering more robust meta-analysis methods that model the binomial distribution of the data, allow for potential between-study heterogeneity in treatment effect, and better account for parameter uncertainty. We show that, despite the visual discrepancy in the Phase II and III summary results, the Phase III trial results for ICH are entirely plausible given full consideration of uncertainty, heterogeneity, and the correct interpretation of a summary meta-analysis result.

### Statistical methods for meta-analysis of Phase II trials

We now suggest methods for meta-analysis of Phase II trials with binary outcomes, and consider issues such as between-trial heterogeneity, zero cells, correct interpretation of summary results, and predicting intervention effects in a subsequent Phase III trial.

### A Bayesian meta-analysis model that accounts for heterogeneity and uncertainty

The fixed-effect approach, as applied by Eikelboom *et al*. [4] to the MI Phase II trials, assumes that all trials are estimating the same common (fixed) intervention effect. In other words, there is no between-trial heterogeneity in the intervention effect and it is only due to chance (sampling error) that the observed trial estimates vary. A general fixed-effect meta-analysis model can be written as follows (Model 1):
1

Here, *Y*_*i*_ is the intervention effect normal estimate (for example the log OR) in trial *i* and *Var*(*Y*_*i*_) is its variance, which is typically assumed to be known (although itself only an estimate) [28]. The model can be estimated using maximum likelihood, and the summary intervention effect estimate () will be a weighted average of the *Y*_*i*_ values, with trial weights equal to the inverse of *Var*(*Y*_*i*_).

There are important drawbacks of model 1, however, in the context of a meta-analysis of Phase II trials. Firstly, as the sample size (and number of events) in each trial is likely to be small the assumption that *Y*_*i*_ has a normal sampling distribution may be inappropriate [9]. Secondly, for each Phase II trial with no events in one of the arms, an arbitrary continuity correction is required in order to obtain *Y*_*i*_ and its variance [29, 30]. Thirdly, and most importantly, the assumption of a fixed intervention effect is unlikely to be realistic, especially if the trials are undertaken in different places and populations, conducted by different researchers (or companies), and with varying lengths of follow-up and implementation (e.g. dose). It is more plausible that the observed intervention effect estimates will vary across trials due to sampling variability (chance) and due to real differences in the intervention effect in each trial.

Therefore, an approach is needed to model the binomial distribution of the data, avoid the need for continuity corrections, and account for between-trial heterogeneity. Furthermore, it is also desirable to account for uncertainty in the estimation of between-trial heterogeneity. We therefore propose that a random-effects logistic regression meta-analysis model is most suitable, within a Bayesian framework [31]. For patient *j* (*j* = 1 to *n*_*i*_) in group *x*_*ij*_ (*x*_*ij*_ = 1 for treatment group, 0 for control group) of trial *i* (*i* = 1 to *k*), the model is (Model 2):


Prior distributions:
2

In model 2, the event outcome status of patient *j* in trial *i* is denoted by *r*_*ij*_, which is 1 if the patient had the event and zero otherwise; *θ*_*i*_ is the true treatment effect (log_e_ OR) in trial *i*, and the *θ*_*i*_ are assumed drawn from a normal distribution with mean *θ* and between-trial variance *τ*^*2*^. The model accounts for the clustering of patients within trials by a separate intercept term, *α*_*i*_, which denotes the baseline (control group) risk for each trial [32]. In model 2, prior distributions must be specified for the unknown parameters (*θ*, *α*_*i*_ and *τ*), which allow other evidence (from outside the trials in the meta-analysis) to be included if available and desired. However, there is often no prior information regarding these unknown parameters, and vague prior distributions are then necessary, such as those shown, with normal prior distributions with large variance given for *θ* and *α*_*i*_. The prior distribution for τ is given as *N*(0,1)*I*(0,), where *I*(0,) indicates the distribution is truncated at zero. This prior distribution is not necessarily ‘vague’ as, for example, it could be made flatter and larger values given more plausibility. However, previous authors have identified that issues arise when the prior distributions for variance parameters are unfeasibly wide [7], and therefore the *N*(0,1)*I*(0,) prior distribution is chosen to reflect a realistic range of plausible values for τ for the MI example. The impact of the choice of prior distributions for *τ*, *θ*, and *α*_*i*_ can be investigated, which is an important consideration in any Bayesian analysis. This is considered further in the Results section.

Posterior estimates of the parameters in model 2 can be obtained using the Gibbs Sampler Markov chain Monte Carlo (MCMC) method [33], which is implemented in WinBUGS version 1.4, Medical Research Council Biostatistics Unit, Cambridge, UK [34] (WinBUGS code is available in Additional file 1: Supporting Information S1). In this article, our model 2 analyses were performed with 100,000 iterations after allowing for a 100,000 iteration burn in, and the samples were thinned by 10 to reduce any concerns of auto-correlation. The convergence of parameters was checked using history and trace plots. The burn in and iteration length were chosen in advance to be large to ensure that the estimation procedure had converged and that the samples fully reflected the posterior distributions, since in the example the trials had small sample sizes and thus wide posterior distributions were expected.

This estimation process enables one to summarize the posterior distribution for the mean intervention effect (*θ*) whilst accounting for the observed binomial data, the posterior distribution of the between-study variance (*τ*^2^), and the prior distributions for *θ* and *τ*. In particular, the mean, median, and 95% credibility intervals can be derived for the mean intervention effect.

### Identifying heterogeneity in Phase II trials: misleading I^2^

To examine heterogeneity, researchers often use the I^2^ statistic, which measures the percentage of variability in intervention effect estimates that is due to between-trial heterogeneity rather than chance (sampling error) [35]. In the MI example, I^2^ is 0% in Phase II meta-analysis for the ICH outcome, and many researchers might therefore conclude that there is no heterogeneity in intervention effects and use a fixed-effect model. However, as Phase II trials are small (for example in terms of outcome events) the variation due to sampling error will be extremely large relative to variation due to between-trial heterogeneity. Thus, regardless of the magnitude of between-trial heterogeneity, the uncertainty due to sampling error will often dominate. Therefore, an I^2^ of 0% (or close to 0%) is potentially misleading, as it may just reflect the trials in the meta-analysis being imprecise. This issue was raised by Higgins and Thompson [35] when they introduced I^2^, and is highlighted in extensive detail by Rucker *et al*. [36].

To address this, we agree with Rucker *et al*. [36] that it is better to evaluate heterogeneity by focusing on the estimate of the between-trial variance (*τ*^2^). Non-zero estimates suggest that heterogeneity is present. However, *τ* will usually be estimated with large uncertainty, and so it may be best to make an *a priori* decision regarding whether to adopt a fixed-effect or random-effects model. As the ultimate aim of a Phase II meta-analysis is to inform a potential Phase III trial, we consider it highly preferable to adopt the random-effects approach by default. As mentioned, this more realistically allows for heterogeneity in intervention effects, and accounting for heterogeneity is an important factor when predicting potential intervention effects in subsequent Phase III trials (see Section “*Using Phase II meta-analysis results to inform Phase III decisions*”).

### Dealing with double zero cells

As discussed, general meta-analysis methods such as model 1 require a continuity correction if there are treatment groups within trials with no events. Using the binomial likelihood within model 2 alleviates this problem for trials where one group has a zero cell [9, 37]. However, with small patient numbers and short follow-up times, Phase II trials may occasionally provide zero events in both treatment groups. In our example, this causes estimation problems for model 2 during the Gibbs sampling estimation of the posterior distributions. To address this, the simplest solution is to exclude any trial with a double zero cell. However we do not advocate this because Phase II trials in the meta-analysis will usually be small, and so even studies with a double zero cell may contribute importantly toward the meta-analysis. Furthermore, they contain valuable information from patients who consented to being included in the trial, and ethically one should ensure their data are included. Therefore, to include trials with double zero cells we applied a continuity correction to them, which thereby avoids the computational issues in WinBUGS. We used the ‘treatment arm’ continuity correction by Sweeting *et al*. [29], which adds 1/(sample size of the opposite treatment group) to each cell in a trial's two by two table, and performs better than the standard approach of adding 0.5, especially when there are imbalances in the sample sizes in each treatment group.

### Using Phase II meta-analysis results to inform Phase III decisions

#### Correct interpretation of summary meta-analysis result

When using the results from a random-effects meta-analysis of Phase II trials to inform Phase III decisions, it is crucial to interpret correctly the summary meta-analysis result () as the estimate of the average intervention effect from the whole distribution of possible effects [5, 8]. The posterior distribution for *θ* therefore reveals the most likely values of, and the uncertainty of, this average intervention effect.

#### Predicting the true intervention effect in a new Phase III trial

When considering whether to conduct a Phase III trial, focusing on the posterior distribution for *θ* may be misleading when heterogeneity in present. The effect in a new trial (*θ*_*i*_) may be very different to the average effect (*θ*), due to the causes of heterogeneity from trial to trial (or setting to setting) [8]. Ideally, the factors causing the heterogeneity would be known so that new trials could focus on implementation strategies (for example doses) and populations most likely to show benefit. However, identifying causes of heterogeneity is problematic if there are few studies (for example fewer than 10) in a meta-analysis and the potential for trial-level confounding. Therefore, we focus here on situations where the Phase II trials in the meta-analysis all include pertinent places, populations, and strategies (such as doses, timing, or length of treatment) for which the intervention effect is of interest.

In this situation, to inform the decision to proceed to Phase III following meta-analysis model 2, one should focus on the predictive distribution for , the intervention effect (log OR) in a new trial that is similar to those already in the meta-analysis:
3

A 95% probability (credibility) interval for  can be obtained by taking the 2.5% and 97.5% values of this distribution. This 95% interval has been referred to as a 95% prediction interval [5, 8], and can be obtained immediately after fitting model 2. As model 2 is a Bayesian framework, the 95% interval will account for the uncertainty in *θ* and *τ*^*2*^ through samples from their posterior distributions. Also, one can use the predictive distribution for  to calculate the probability that the intervention will be truly effective in the new trial [38], either at all (probability(new OR <1)) or by some clinically relevant amount, such as the odds being reduced by at least 10% (probability(new OR <0.9)).

#### Predicting the chance of success in a Phase III trial with a given sample size

Though the true intervention effect () is of fundamental interest, a more pertinent question facing Phase III funders is: what is the probability that the intervention will be identified as beneficial in a new trial with a given sample size? To help answer this, during the estimation of model 2 one can also derive an approximate predictive distribution for the intervention effect estimate, , in a new trial of particular sample size, :
4

where  is the intervention effect in a new trial. The variance of  must be specified by the user, as it accounts for the additional uncertainty that arises from the sampling error in the new trial of a particular sample size and event risk. In this article, to specify the variance we utilise the well-known approximate formula for the variance of:
5

where  and  are the number of events in the new trial’s experimental groups and control groups, respectively,  and  are the number of non-events in the new trial’s experimental and control groups, respectively, and the total sample size is . This calculation of the variance mimics how it will be obtained when a new trial is done, as the formula is based on the frequentist estimation, which is the standard approach to analyze Phase III trials. At each iteration of the model estimation, the values of  and  are thus needed in order to derive the variance for each  sampled during the estimation process. We consider two options to achieve this here. Option 1 is to fix the baseline risk ( and ) and sample size in each group, which allows  to be obtained for each  sampled, and thus the variance of  is then known. Option 2 is to assume a fixed variance of  regardless of the actual value of  sampling, again based on assuming particular sample sizes and event risks in both groups. The full details of these options are provided in Additional file 1: Supporting Information S2.

Implementing options 1 or 2 allow for an approximate 95% probability interval for  to be calculated every time  is sampled, by:
6

Therefore, across all samples during the estimation process, one can also derive predictive distributions for the lower and upper bounds of the 95% interval for . One can then calculate probabilities to inform Phase III decisions. In particular the probability that, in a new trial with a sample size of  and a control group risk of , the upper bound of the 95% interval for  will be lower than 0 (that the lower bound of the CI for the OR will be <1). In other words, the probability that the new trial will identify the intervention as effective by the entire 95% interval for the OR being in favor of the intervention.

## Results

### Application to the bolus thrombolytic therapy trials

We now consider the aforementioned statistical methods and issues in relation to the thrombolytic therapy trials introduced in the Methods section.

### Misleading I-squared

I^2^ is 0 and 8% for the ICH and reinfarction outcomes, respectively. Therefore, it might appear that there is very little between-trial heterogeneity in the effect of bolus therapy for both outcomes. However, after fitting the Bayesian random-effects logistic regression in model 2, the posterior distribution for τ has a median value of 0.66 and a 95% credible interval of 0.04 to 1.91 for ICH. Similarly, for reinfarction, the median estimate for τ is 0.28 and has a 95% credible interval of 0.01 to 0.93. This suggests that τ is not zero for either outcomes and thus, in contrast to the initial conclusion from I^2^, heterogeneity does seem to exist and may even be substantial. This highlights how I^2^ can be misleading when the included trials are small [36].

### Fixed-effect versus random effects results

As mentioned, application of model 2 to the data handles all studies that had one zero cell, but required the continuity correction of Sweeting *et al*. [29] in the study containing a zero in two cells. The meta-analysis results are shown in Table 2. The impact of this double zero study on the meta-analysis conclusion was negligible; compared to an analysis that excluded the study, the means and medians of all posterior distributions were very similar and standard deviations were only reduced at the third decimal place.

**Table 2:** **Meta-analysis results for ICH and reinfarction from a Bayesian model and the original frequentist approach**

Outcome	Summary OR	95% CrI/CI for summary OR	(95% CrI)	Probability summary OR <1	Probability true OR <1 in new trial
**ICH**					
Bayesian	0.485	0.155 – 1.266	0.660 (0.043 – 1.914)	0.937	0.824
Original	0.552	0.287 – 1.063	-	-	-
**Reinfarction**					
Bayesian	0.773	0.502 – 1.179	0.276 (0.013 – 0.929)	0.901	0.787
Original	0.779	0.568 – 1.066	-	-	

Phase II meta-analysis results for ICH and reinfarction from a Bayesian random-effects logistic regression model (model 2) and from the original frequentist fixed-effect approach of Eikelboom *et al*. [4]. CI, confidence interval; ICH, intracranial hemorrhage; OR, odd ratio; CrI credible interval.

The frequentist fixed-effect analysis results of Eikelboom *et al*. [4] are compared to the Bayesian random-effects model 2 results in Figure 1 and Figure 2, for outcomes ICH and reinfarction respectively. For both approaches, the summary ORs are in favor of bolus therapy (summary OR <1). However, the fixed-effect meta-analysis gives 95% confidence intervals that are much narrower than the 95% credible intervals from the random-effects model, as the latter more appropriately accounts for heterogeneity and parameter uncertainty. For example, for ICH the 95% confidence interval for the summary OR is 0.29 to 1.06 from the fixed-effect analysis, and the 95% credible interval is 0.16 to 1.27 from the random-effects analysis. The 95% credible intervals are wide, reflecting large uncertainty in the summary intervention effect from the random-effects analysis. This is unsurprising given the Phase II trials being synthesized have small sample sizes and heterogeneity in the intervention effect estimate. However, the majority of the intervals are below 1 (in favor of bolus therapy).

### Inferences for the predicted true intervention effect in a new Phase III trial

**Figure 3:**
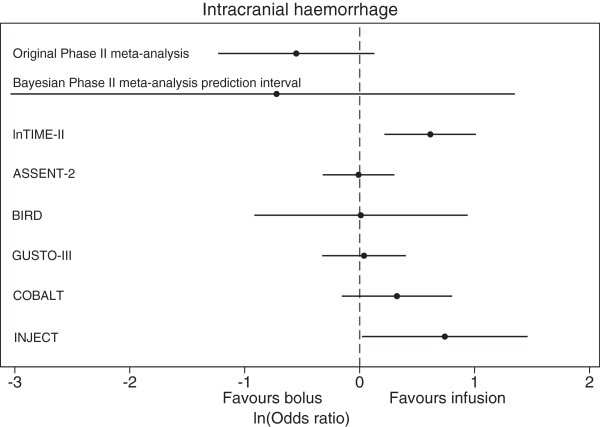
**Comparison of Bayesian prediction interval from Phase II meta-analysis with original meta-analysis and subsequent Phase III trials for ICH.** lnTIME-II, ASSENT-2, BIRD, GUSTO-III, COBALT and INJECT are the six subsequent Phase III trials, which compared bolus to infusion therapy for patients with an acute myocardial infarction. Horizontal lines indicate the 95% confidence intervals for the estimated log ORs in the Phase III trials and the original Phase II meta-analysis, and the 95% prediction interval in the Bayesian Phase II meta-analysis [22–27].

Following model 2, the 95% prediction interval for the true OR in a new trial can be calculated from the predictive distribution for  (Equation 3). For ICH, this is calculated to be 0.05 to 3.79 (Figure 3), and for reinfarction this is calculated to be 0.29 to 2.04. These prediction intervals are both much wider than the 95% credible intervals for the summary (average) intervention effects for each outcome, as they reveal the wider range of intervention effects across settings and populations due to heterogeneity. Crucially these intervals overlap an OR of 1, and therefore in some settings we cannot rule out that bolus therapy may not be effective. However, the majority of the prediction intervals are below 1. This can be quantified more formally by calculating the proportion of the predictive distributions for  that is below 0 (OR <1). This gives the probability that bolus therapy will be more effective than control in a new trial, and is 0.824 for ICH and 0.787 for reinfarction. These reasonably large probabilities suggest that the therapy has potential clinical value and that Phase III trials are worth considering.

### Probability of success in a new trial with a given sample size

Given that bolus therapy has large probability of being truly effective in a new trial, funders next need to consider whether a Phase III trial is likely to show this statistically. For simplicity, consider just ICH and let us calculate the probability that, for a trial with a given sample size*,* the derived 95% interval for the OR will have an upper bound less than 1. We consider both options 1 and 2 for obtaining the variance of  to derive this interval.

**Figure 4:**
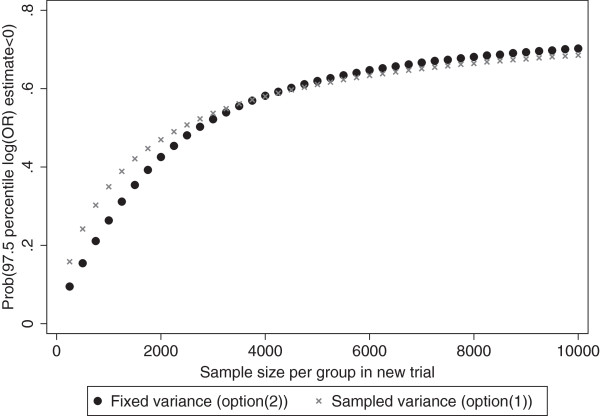
**Probability that the upper bound of the OR's 95% credible interval may be less than 1 in a new trial for ICH.** Fixed variance (option 2) indicates the variance of  is assumed to be a fixed value which is approximated by assuming a baseline risk of 0.01 in the control group, a particular sample size, and a treatment effect of 0.485 (summary OR estimate from model 2). Sampled variance (option 1) indicates the variance of  is calculated for each sample of the estimation process, assuming a baseline risk of 0.01 in the control group, a particular sample size, and using the sampled ( (Equation 6).

Let us assume a control group risk of 0.01 for ICH in the new trial (a plausible baseline risk from previous trials [4]), which is the probability of an ICH event in the infusion therapy group. Under this assumption, the probability that bolus therapy will be shown to be effective in the new trial is illustrated in Figure 4, for varying chosen sample sizes and for each of options 1 and 2. As the sample size increases, the probability of success in a new trial also increases, which reflects the narrower credible intervals that arise from larger patient numbers. Options 1 and 2 give reasonably similar results.

When the sample size is unrealistically large (10,000,000 patients per arm), such that the trial is tending toward an infinite sample size, the probability of success tends to the probability that exp() is less than one, which equals 0.824 as noted above. For more realistic sample sizes, the probability of success is much lower. For example, with 2,000 patients in each arm of the trial the probability of success is only about 0.4. However, increasing to 4,000 patients per arm increases the success probability to about 0.6. In this manner, Figure 4 reveals to funders how much is gained (in terms of success probability) by increasing the sample size. They can then weigh this gain against the increased costs needed to recruit more individuals.

### Comparison with subsequent randomized Phase III trials

As introduced in the Methods section, Eikelboom *et al*. [4] conclude that the meta-analysis results are contradictory for Phase II and subsequent Phase III trials for ICH (Figure 1 and Table 2), as their summary results are in opposite directions with very little overlap in their confidence intervals; Phase II trials favor bolus therapy, whereas Phase III trials favor infusion therapy. However, their comparison was inappropriate, as their analysis ignored heterogeneity in the treatment effect. Indeed, the apparent disagreement in their Phase II and III summary results is potentially resolved when considering the 95% prediction interval for the OR in a new trial that can be obtained from our Phase II trial meta-analysis. As noted above, this 95% prediction interval is 0.05 to 3.79 and is wide due to the large heterogeneity and uncertainty present. This interval includes all the estimates of treatment effect for ICH obtained from the subsequent Phase III trials (Figure 3), suggesting that Eikelboom *et al*. [4] were incorrect as the Phase III results are plausible given the Phase II evidence. It is conceivable that the settings and populations of subsequent Phase III trials related more to those effects towards the upper side of the 95% prediction interval.

### Choice of prior distribution for between-trial variance

**Figure 5:**
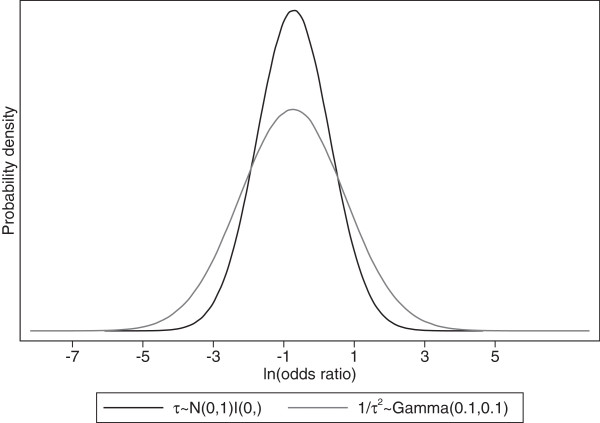
**Posterior distribution for treatment effect in a new trial (**  **for intracranial hemorrhage assuming two different priors for the heterogeneity parameter.**

The choice of vague prior distribution for the between-trial variance (*τ*^*2*^) in model 2 is not a trivial decision [7, 39], and may influence the posterior inferences. Table 3 shows the summary estimates and 95% prediction intervals for the OR for ICH in a new study, as obtained from model 2 and Equation 3 using a variety of different prior distributions. Figure 5 shows the posterior distributions for  for priors 2 and 6 in Table 3. The summary treatment effect estimate is similar regardless of the prior chosen. However, the width of the posterior distribution for the treatment effect is vulnerable to the choice of prior, and this affects the 95% prediction intervals. Where possible, external evidence regarding the between-study heterogeneity may be useful to include within the prior distribution to ensure vague but realistic prior distributions are chosen as discussed [6].

**Table 3:** **Sensitivity to prior distribution for between-trial variance in prediction interval for treatment effect for ICH**

Prior distribution	OR	95% prediction interval OR	(95% CrI)	Probability OR in new trial <1
1: τ ~ Uniform(0,2)	0.470	0.032 - 5.751	0.915 (0.065 - 1.920)	0.788
2: 1/τ^2^ ~ Gamma(0.1, 0.1)	0.465	0.021 - 7.938	0.825 (0.244 - 3.146)	0.787
3: Log(τ^2^) ~ Uniform(-10,1.386)	0.499	0.094 - 2.097	0.139 (0.008 - 1.654)	0.907
4: τ^2^ ~ Uniform(0.001,4)	0.449	0.020 - 8.652	1.257 (0.293 - 1.959)	0.741
5: 1/ τ^2^ ~ Pareto(1,0.25)	0.447	0.020 - 8.496	1.260 (0.293 - 1.960)	0.742
6: τ ~ Normal(0,1)*I*[0,]	0.485	0.049 - 3.793	0.657 (0.037 - 1.911)	0.824

ICH intracranial hemorrhage; OR odds ratio; CrI credible interval.

### Adjusting for potential optimism in Phase II results

The estimates of the OR in the individual Phase III trials for ICH and reinfarction are closer to one when compared to most of those from the individual Phase II trials. As shown, this is plausibly due to the heterogeneity. However, as Eikelboom *et al*. [4] discuss, it may also be due to optimism and bias in the Phase II trials. Indeed it is common in medical research for interventions to show early promise, only for subsequent large studies to show no or lower benefit [40]. For this reason, following a meta-analysis of Phase II trials, it may be important to account for potential optimism when predicting the treatment effect in subsequent Phase III trials.

### Examining potential publication bias

**Figure 6:**
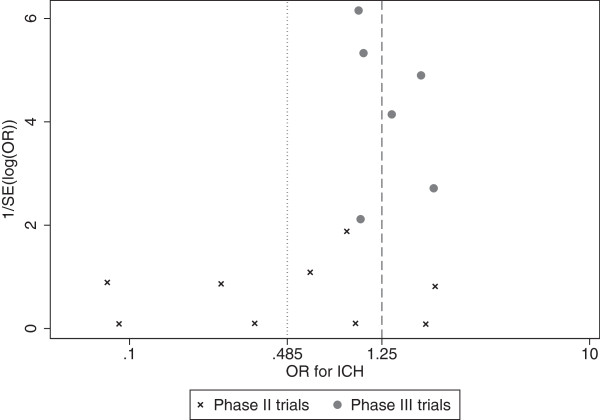
**Funnel plot of 1/SE(log(OR)) versus OR for ICH in Phase II and Phase III trials.** Dotted line represents the summary treatment effect estimate for ICH from the Bayesian random-effects logistic regression analysis (model 2) of the Phase II trials; dashed line indicates the summary treatment effect estimate for ICH by Eikelboom *et al*. [4] for the Phase III trials. The OR axis is shown on the log-scale. OR odds ratio; ICH intracranial hemorrhage, SE standard error.

One cause for potential optimism may be publication bias, which is an issue that occurs when trials with more favorable results are more likely to be published than those with less favorable results [41]. Publication bias can be explored using funnel plots where, if there is no evidence of publication bias, the assumption is that the trials should be symmetrically distributed about the estimates from larger studies, in a funnel-like shape. A funnel plot of only the Phase II trials for ICH in Figure 6 suggests that there is no clear evidence of publication bias since the observed estimates appear equally spread in both directions around the estimates from the largest Phase II trials. This contradicts the asymmetric funnel plot for ICH shown by Eikelboom *et al.* [4] (Figure 6), which displayed both Phase II and Phase III trials. This suggests asymmetry in their plots may have been caused by heterogeneity rather than genuine publication bias [42]. The funnel plot for reinfarction (not shown) in the Phase II trials also shows no clear evidence of asymmetry.

### Including skeptical prior distributions to adjust for optimism

**Figure 7:**
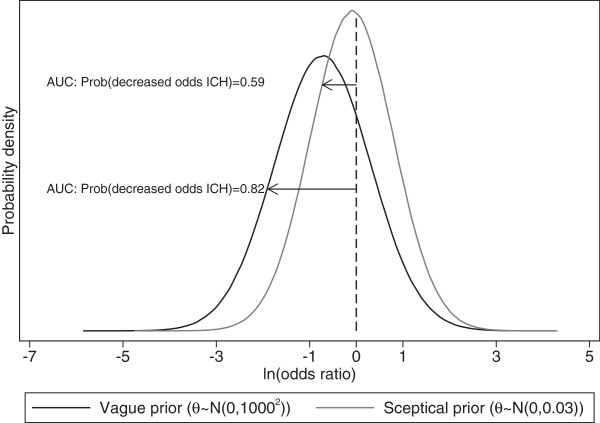
**Posterior distributions for treatment effect (log(OR)) in a new trial for ICH assuming a skeptical and vague prior distribution for log(OR).** Vague prior distribution (θ ~ N(0,1000^2^)) and skeptical prior distribution (θ ~ N(0,33.33)) for the log OR. The area under the curve (AUC) that is less than zero is the probability that bolus therapy will truly be effective in a new trial. ICH intracranial hemorrhage; AUC area under the curve.

Assessment of potential publication bias is difficult, and usually at least 10 studies are recommended [43]. Even if there is no clear evidence of publication bias, Phase II trials may be more prone to bias in their design, execution, and analysis, which could also cause optimistic meta-analysis results for Phase II trials. It is possible to limit the potential optimism in the Bayesian analysis by using a realistic or ‘skeptical’ prior distribution for the pooled intervention effect that does not allow large intervention effect sizes [6, 40]. Caution must be taken when deriving a skeptical prior distribution as there is a danger of using an informative prior not based on evidence of plausibility. Therefore clinical guidance is needed, or evidence from external trials can be used, to inform a plausible magnitude of treatment effect. For example, the external trial information could come from a trial where a similar treatment was evaluated (such as a drug from the same class), but perhaps in a different disease area or patient group. Spiegelhalter *et al*. [6] discuss how to mathematically derive a skeptical prior distribution based on plausible treatment differences where there is only a small probability that the treatment effect is as large as the alternative hypothesis. For example, a skeptical prior distribution on the summary OR could be such that there is little chance (say just 5%) that the experimental treatment would reduce the odds of the event of interest by more than, say, 25% compared to the control treatment. This could relate to the summary log OR having a prior Normal distribution, with mean zero and variance 0.03. Figure 7 shows how this skeptical prior distribution for *θ* alters the posterior distribution for the intervention effect ( ) in a new trial for ICH, compared to the original vague prior distribution for *θ* in model 2. The posterior distribution is drawn closer to zero, and consequently, the probability that the estimated OR is less than 1 in a new trial is now lower. It should be noted that the use of skeptical prior distributions may not be necessary in all meta-analyses of Phase II trials; it will depend on factors such as the perceived quality (risk of bias) of the available Phase II trials, and whether the meta-analysis results otherwise appear optimistic relative to evidence of the effectiveness of related interventions in the same or related disease area.

## Discussion

The decision to progress to Phase III is based on all existing evidence, which includes information other than the results of Phase II trials, such as costs and feasibility. However, if multiple Phase II trials exist, such as in the example by Eikelboom *et al.* [4] in this paper and others identified by the Cochrane Collaboration (such as [44]), a meta-analysis of the Phase II trials should be considered important. The example in this paper has illustrated that meta-analysis of Phase II trials can be useful to inform Phase III trial decisions. We have tackled a number of methodological issues that arise when conducting a meta-analysis of Phase II trials. In particular, the choice of meta-analysis model, how to deal with heterogeneity [5] and zero cells [29], and how to translate the meta-analysis results to inform new studies. Sutton *et al*. [10] have also considered the use of meta-analysis to inform the sample size of future trials (but not in the context of Phase II and III) and mainly in relation to how updated meta-analysis results could change after the new trial is performed.

Heterogeneity is a genuine problem in meta-analysis and to ensure the Phase II meta-analysis is relevant to Phase III decisions, we recommend that heterogeneity is reduced by only including those Phase II trials that are relevant to the populations and settings for which the intervention is intended. It is difficult to examine and quantify the potential heterogeneity in a meta-analysis of Phase II trials due to the small number of studies and the small number of patients within studies, which can cause low power and large within-study variation. The I^2^ statistic is always likely to be small when within-study variances are large, as shown for the ICH outcome [36]. Since Phase II trials have small patient numbers and are often conducted separately, we believe it is likely that heterogeneity exists and so should be accounted for. Therefore, researchers may decide *a priori* that a random-effects model will be used for the meta-analysis, and thereby avoid reliance on I^2^.

When informing Phase III decisions, we have shown the importance of deriving prediction intervals for the true intervention effect in a new trial [5] and, perhaps most pertinently, the probability of observed success for a new trial with a given sample size. These are more meaningful than the summary meta-analysis result itself, which relates only to the average effect [8]. The Bayesian framework naturally incorporates heterogeneity and parameter uncertainty, which means that posterior distributions for the intervention effect in a new trial reflect the uncertainty in potential Phase III trial results. Bayesian meta-analysis methods lead naturally to direct probability statements, and can also limit potential bias and optimism in the prediction intervals from Phase II trials through skeptical prior distributions [6]. However, the choice of prior distribution for heterogeneity can influence the results [7, 39] and therefore sensitivity to the choice of prior distribution is recommended.

We envisage that, in most situations, a meta-analysis of Phase II trials is likely to reveal the large uncertainty upon which the Phase III trial decision is based, even despite results of the individual trials being pooled. The small sample sizes in Phase II trials, and the rare event rate in these particular trials, combined with between-trial heterogeneity in intervention effects, are the key contributing factors to the large uncertainty. This makes the posterior distribution (and 95% prediction intervals) wide, but this is merely a full reflection of the information available and will ensure funders are fully aware of statistical uncertainty when making their decisions for Phase III. Funders can improve their chances of a Phase III success by increasing sample sizes (Figure 4), but this causes an increase in trial costs. Other considerations away from statistical uncertainty are also crucial of course, such as the biological understanding of a drug's mechanism, the acceptability of the intervention of interest, and the market demand for the intervention. Therefore Phase III predictions should be just one, albeit important, part of the decision-making process.

### Relevance of our work to recent meta-analyses of Phase II trials

In this paper, we focused on improving the meta-analysis of Phase II trials conducted by Eikelboom *et al*. [4], in which they ignored heterogeneity by using a fixed-effect model, and did not model directly the binomial distribution of the data. We also identified other examples, in more recent years, where the method for meta-analyzing Phase II studies could be improved similarly. In particular, the decision to use a fixed-effect or random-effects model is often based on the *P* value derived from the Q statistic (chi-squared test for heterogeneity [3]) and/or the I^2^ statistic [45–48]. If the *P* value from the chi-squared test is not statistically significant, and/or I^2^ is low, a fixed-effect model is often used. However, with few studies there is very low power to detect heterogeneity, and therefore a significant *P* value is unlikely in the meta-analysis of Phase II trials and so genuine heterogeneity may be ignored. Similarly, we showed low values of I^2^ are also potentially misleading for Phase II meta-analysis.

We are aware of two meta-analyses of Phase II trials where authors decided *a priori* that a random-effects model was more appropriate because of the expectation that the studies would estimate different, yet related, treatment effects [44, 49]. This approach concurs with our recommendation above. However, in these and other articles using a random-effects model, the conclusions only focused on the pooled estimate of the average treatment effect, and the prediction interval for the treatment effect in a new trial was not considered [44–49]. Thus, the full uncertainty of the potential treatment effect in new populations (or Phase III studies) is often ignored. Finally, it is also common for meta-analyses of Phase II trials to pool treatment effects using the inverse variance method (model 1), rather than modelling the binomial distribution of the data more exactly as shown in model 2 [44–49].

## Conclusions

The choice of meta-analysis methods can influence the decision about whether to proceed to Phase III and thus the methods need to be clearly documented and investigated whenever a Phase II meta-analysis is performed. Eikelboom *et al.* [4] originally conducted a fixed-effect meta-analysis of Phase II trials and compared the results to a meta-analysis of subsequent Phase III trials. They concluded that there were conflicting results between the two meta-analyses for ICH. However, our Bayesian random-effects logistic regression analysis with estimated prediction intervals shows that the results are not necessarily contradictory.

### Recommendations for good practice

Table 4 summarizes our recommendations for good practice within meta-analysis of Phase II trials.

**Table 4:** **Recommendations for improved meta-analysis of Phase II trials of binary outcomes**

Issue	Recommendation
Framework	Use a logistic regression model to model the binomial distribution of the data within studies, and to avoid continuity corrections given a zero event in one arm.
Choice of model	Do not make decisions to use a fixed-effect or random-effects model based on I^2^ or tests for heterogeneity.
Heterogeneity	State *a priori* that a random-effects model will be used to account for heterogeneity in treatment effects.
Uncertainty	Use a Bayesian framework to account for all parameter uncertainty and external evidence (such as the between-study variance) and to enable direct probabilistic inferences. However, a sensitivity analysis to the choice of prior distributions is required.
Prediction intervals	Report 95% prediction intervals as they reveal the potential treatment effect in a new population, and inform subsequent Phase III decisions.
Bias	Use skeptical prior distributions for the treatment effect if there is evidence to suggest the Phase II trials may be biased in favor of the treatment.

## Electronic supplementary material

Additional file 1: **Supporting Information.** (DOCX 29 KB)

## Abbreviations

OR: Odds ratio
ICH: Intracranial hemorrhage
MI: Myocardial infarction
MCMC: Markov chain Monte Carlo
CI: Confidence interval
CrI: Credible interval
SE: Standard error
AUC: Area under curve.
