# Supplementary material for: Meta-analysis of randomized phase II trials to inform subsequent phase III decisions
Source: Trials. 2014 Sep 3;15:346. doi: 10.1186/1745-6215-15-346 (PMC4162965; doi:10.1186/1745-6215-15-346)
Supplement: Supplementary file 1 — Additional file 1: Supporting Information. (DOCX 29 KB) [file 13063_2014_2218_MOESM1_ESM.docx]

Supporting Information

**S1: WinBUGS code for Bayesian random-effects logistic regression model (2)**

Model {

for( i in 1 : Nstud ) {

*# Events on infusion therapy in trial i*

rI[i] ~ dbin(pI[i], nI[i])

*# Events on bolus therapy in trial i*

rB[i] ~ dbin(pB[i], nB[i])

*# Log odds of event on infusion therapy in trial i*

logit(pI[i]) <- mu[i]

*# Log odds of event on bolus therapy in trial i*

logit(pB[i]) <- mu[i] + delta[i]

*# Vague normal prior distribution for log odds of event on infusion therapy in trial i*

mu[i] ~ dnorm(0.0,1.0E-5)

*# Normal distribution for log odds ratio in trial i with mean d and variance=1/prec*

delta[i] ~ dnorm(d, prec)

}

or <- exp(d)

*# Vague normal prior distribution for average log odds ratio*

d ~ dnorm(0.0,1.0E-6)

*# Vague normal prior distribution for between-trial standard deviation truncated at zero*

tau~dnorm(0,1)I(0,)

tau.sq<-tau*tau

prec<-1/(tau.sq)

*# Distribution for true log odds ratio in a new trial*

d.new ~ dnorm(d, prec)

or.new <-exp(d.new)

*# Probability that the average odds ratio is less than 1*

prob1 <- step(1-or)

*# Probability that the true odds ratio in a new trial is less than one*

prob1.new <- step(1-or.new)

*# Distribution for log odds ratio in a new trial for ICH for varying sample sizes*

for (k in 1:K) {

y.new[k] ~ dnorm(d.new,prec2[k])

ory.new[k] <- exp(y.new[k])

*# Estimation of variance of y.new*

prec2[k]<-1/((1/a[k])+(1/b[k])+(1/c[k])+(1/di[k]))

a[k]<-(exp(d.new)*n[k]*c[k])/(di[k]+(exp(d.new)*c[k]))

b[k]<-n[k]-a[k]

*# Probability OR in new trial is less than one*

prob1.orynew[k]<-step(1-ory.new[k])

*# Upper bound of approximate 95% interval for y.new*

up.ynew[k] <- y.new[k]+1.96*sqrt(1/prec2[k])

*# Probability that approximate upper bound of 95% interval for y.new is less than 1*

sig.ynew[k] <- step(0-up.ynew[k])

}}

**S2: How to estimate the variance of the intervention effect estimate,** $\boldsymbol{Y}_{\boldsymbol{i}_{\boldsymbol{new}}}$**, in a new trial of a particular sample size,** $\boldsymbol{N}_{\boldsymbol{i}_{\boldsymbol{new}}}$**.**

From model (2), it is possible to derive an approximate predictive distribution for the intervention effect estimate, $Y_{i_{new}}$, in a new trial of particular sample size, $N_{i_{new}}$:

|  | $Y_{i_{new}}\sim N\left( \theta_{i_{new}},var(Y_{i_{new}}) \right)$ | (6) |
| --- | --- | --- |

In order to estimate the variance of $Y_{i_{new}}$, there are the following two options:

***Option 1: Estimate the variance of*** $Y_{i_{new}}$ ***in each drawn sample, assuming a fixed baseline risk and sample size***

First, assume a fixed proportion of events (baseline risk) in the control group in the new trial; this could be chosen to reflect the baseline risk in the intended population. Thus $\frac{c_{i_{new}}}{c_{i_{new}}+d_{i_{new}}}$ is specified. Then also specify the sample size of the control group (${nc}_{i_{new}}$*=*$c_{i_{new}}+d_{i_{new}}$). The chosen baseline risk and sample size thereby fix $c_{i_{new}}$ and$d_{i_{new}}$. Also, specify the treatment group sample size (${nt}_{i_{new}}$*=*$a_{i_{new}}+b_{i_{new}}$). Then, for each $Y_{i_{new}}$ that is sampled during the estimation process, $a_{i_{new}}$ and $b_{i_{new}}$ can be derived as follows:

|  | $\theta_{i_{new}}=\log\left( \frac{a_{i_{new}}d_{i_{new}}}{b_{i_{new}}c_{i_{new}}} \right)$  $\Rightarrow a_{i_{new}}=\frac{exp{\left( \theta_{i_{new}} \right)nt}_{i_{new}}c_{i_{new}}}{d_{i_{new}}+exp\left( \theta_{i_{new}} \right)c_{i_{new}}}$  ${\Rightarrow b}_{i_{new}}={nt}_{i_{new}}-a_{i_{new}}$ | (7) |
| --- | --- | --- |

In equation (7),$\boldsymbol{nt}_{\boldsymbol{i}_{\boldsymbol{new}}}$,$\boldsymbol{c}_{\boldsymbol{i}_{\boldsymbol{new}}}$ and$\boldsymbol{d}_{\boldsymbol{i}_{\boldsymbol{new}}}$ are fixed (specified in advance), and $\boldsymbol{\theta}_{\boldsymbol{i}_{\boldsymbol{new}}}$is the current sampled intervention effect for the new trial. In this way, each $\boldsymbol{Y}_{\boldsymbol{i}_{\boldsymbol{new}}}$ that is sampled has a variance approximated by$\frac{\mathbf{1}}{\boldsymbol{a}_{\boldsymbol{i}_{\boldsymbol{new}}}}\mathbf{+}\frac{\mathbf{1}}{\boldsymbol{b}_{\boldsymbol{i}_{\boldsymbol{new}}}}\mathbf{+}\frac{\mathbf{1}}{\boldsymbol{c}_{\boldsymbol{i}_{\boldsymbol{new}}}}\mathbf{+}\frac{\mathbf{1}}{\boldsymbol{d}_{\boldsymbol{i}_{\boldsymbol{new}}}}\mathbf{,}$ where $\boldsymbol{c}_{\boldsymbol{i}_{\boldsymbol{new}}}$ and $\boldsymbol{d}_{\boldsymbol{i}_{\boldsymbol{new}}}$are chosen and $\boldsymbol{a}_{\boldsymbol{i}_{\boldsymbol{new}}}$ and $\boldsymbol{b}_{\boldsymbol{i}_{\boldsymbol{new}}}$ are obtained from equation (7) for that sample.

*Option 2: Assume a fixed variance of* $\boldsymbol{Y}_{\boldsymbol{i}_{\boldsymbol{new}}}$

A simpler, but potentially less accurate approach than option (1), is to specify a fixed variance of$Y_{i_{new}}$, regardless of the $\theta_{i_{new}}$ sampled. To do this, in addition to specifying $c_{i_{new}}$ and$d_{i_{new}}$ based on the assumed baseline risk and control group sample size (see option (1)), one needs to also fix $a_{i_{new}}$and$b_{i_{new}}$. One therefore needs to assume $\theta_{i_{new}}$ is fixed at some value (=$\log\left( \frac{a_{i_{new}}d_{i_{new}}}{b_{i_{new}}c_{i_{new}}} \right)$). For example, one might fix $\theta_{i_{new}}$ to be the mean of the posterior distribution for$\theta_{i_{new}}$. Then $a_{i_{new}}$ and $b_{i_{new}}$ can be obtained.
